# Iduronate-2-sulfatase transport vehicle rescues behavioral and skeletal phenotypes in a mouse model of Hunter syndrome

**DOI:** 10.1172/jci.insight.145445

**Published:** 2021-10-08

**Authors:** Annie Arguello, René Meisner, Elliot R. Thomsen, Hoang N. Nguyen, Ritesh Ravi, Jeffrey Simms, Iris Lo, Jessica Speckart, Julia Holtzman, Thomas M. Gill, Darren Chan, Yuhsiang Cheng, Chi-Lu Chiu, Jason C. Dugas, Meng Fang, Isabel A. Lopez, Hilda Solanoy, Buyankhishig Tsogtbaatar, Yuda Zhu, Akhil Bhalla, Kirk R. Henne, Anastasia G. Henry, Anthony Delucchi, Simona Costanzo, Jeffrey M. Harris, Dolores Diaz, Kimberly Scearce-Levie, Pascal E. Sanchez

**Affiliations:** 1 Denali Therapeutics Inc., South San Francisco, California, USA.; 2 Behavioral Core, Gladstone Institute of Neurological Disease, San Francisco, California, USA.

**Keywords:** Neuroscience, Therapeutics, Behavior, Neurological disorders, Translation

## Abstract

Mucopolysaccharidosis type II (MPS II) is a lysosomal storage disorder caused by deficiency of the iduronate-2-sulfatase (IDS) enzyme, resulting in cellular accumulation of glycosaminoglycans (GAGs) throughout the body. Treatment of MPS II remains a considerable challenge as current enzyme replacement therapies do not adequately control many aspects of the disease, including skeletal and neurological manifestations. We developed an IDS transport vehicle (ETV:IDS) that is engineered to bind to the transferrin receptor; this design facilitates receptor-mediated transcytosis of IDS across the blood-brain barrier and improves its distribution into the brain while maintaining distribution to peripheral tissues. Here we show that chronic systemic administration of ETV:IDS in a mouse model of MPS II reduced levels of peripheral and central nervous system GAGs, microgliosis, and neurofilament light chain, a biomarker of neuronal injury. Additionally, ETV:IDS rescued auricular and skeletal abnormalities when introduced in adult MPS II mice. These effects were accompanied by improvements in several neurobehavioral domains, including motor skills, sensorimotor gating, and learning and memory. Together, these results highlight the therapeutic potential of ETV:IDS for treating peripheral and central abnormalities in MPS II. DNL310, an investigational ETV:IDS molecule, is currently in clinical trials as a potential treatment for patients with MPS II.

## Introduction

Mucopolysaccharidosis type II (MPS II), also known as Hunter syndrome, is an X-linked recessive lysosomal storage disorder (LSD) caused by deficiency in iduronate-2-sulfatase (IDS) activity, an enzyme responsible for the catabolism of glycosaminoglycans (GAGs) (1, 2). Inactivity, insufficiency, or absence of IDS leads to widespread accumulation of GAGs and subsequent lysosomal dysfunction in multiple organs and tissues, including the central nervous system (CNS) (3, 4). The resulting clinical presentation of MPS II typically includes coarse facial features, hepatosplenomegaly, joint and skeletal involvement, cardiopulmonary disease, hearing loss, and dysfunction in the central and peripheral nervous system (2, 5). Approximately two-thirds of MPS II patients display the neuronopathic form of the disease, which, in addition to earlier presentation of the somatic disease, is characterized by progressive debilitating neurocognitive deficits. The age of symptom onset is variable but typically occurs before 3 years of age and manifests as a plateau of cognitive and adaptive development, followed by progressive neurocognitive regression (5–7). Currently available enzyme replacement therapies (ERTs) for MPS II have been successful at reducing the accumulation of GAGs in the periphery (8, 9). However, these therapies do not adequately control many aspects of the disease, including skeletal, cardiac, and pulmonary involvement, and, most importantly, do not cross the blood-brain barrier (BBB) to effectively treat the neuronopathic manifestations of MPS II (8, 9).

We recently described a novel ERT, which consists of an IDS enzyme fused to an engineered transferrin receptor–binding (TfR-binding) Fc fragment (ETV:IDS) (10, 11). ETV:IDS was developed to exploit receptor-mediated transcytosis at the BBB and increase delivery of IDS to the brain parenchyma, where it is predicted to treat neurological symptoms of MPS II while maintaining therapeutic benefit in the periphery. Improved brain delivery of IDS using our ETV platform translated to a reduction in accumulated GAGs in the CNS and periphery in a mouse model of MPS II and resulted in changes in multiple disease-related CNS biomarkers downstream of GAG accumulation in the brain, including lysosomal dysfunction, neuroinflammation, and neuronal injury (11).

Here we evaluated multiple behavioral domains to assess cognitive and motor deficits in our mouse model of MPS II, which combines genetic deletion of the IDS gene with expression of a chimeric human/mouse TfR (*Ids*-KO TfR^mu/hu^). In addition, we evaluated auricular and skeletal manifestations in these mice using histological and micro-CT analysis, respectively. We further demonstrate that systemic administration of ETV:IDS is efficacious in rescuing MPS II–relevant neurobehavioral and skeletal abnormalities, highlighting the therapeutic potential of ETV:IDS to improve aspects of MPS II disease that are minimally affected by currently available ERTs.

## Results

### Systemic administration of ETV:IDS reduces CNS GAGs, microgliosis, and neurofilament light chain levels.

**Figure 1:**
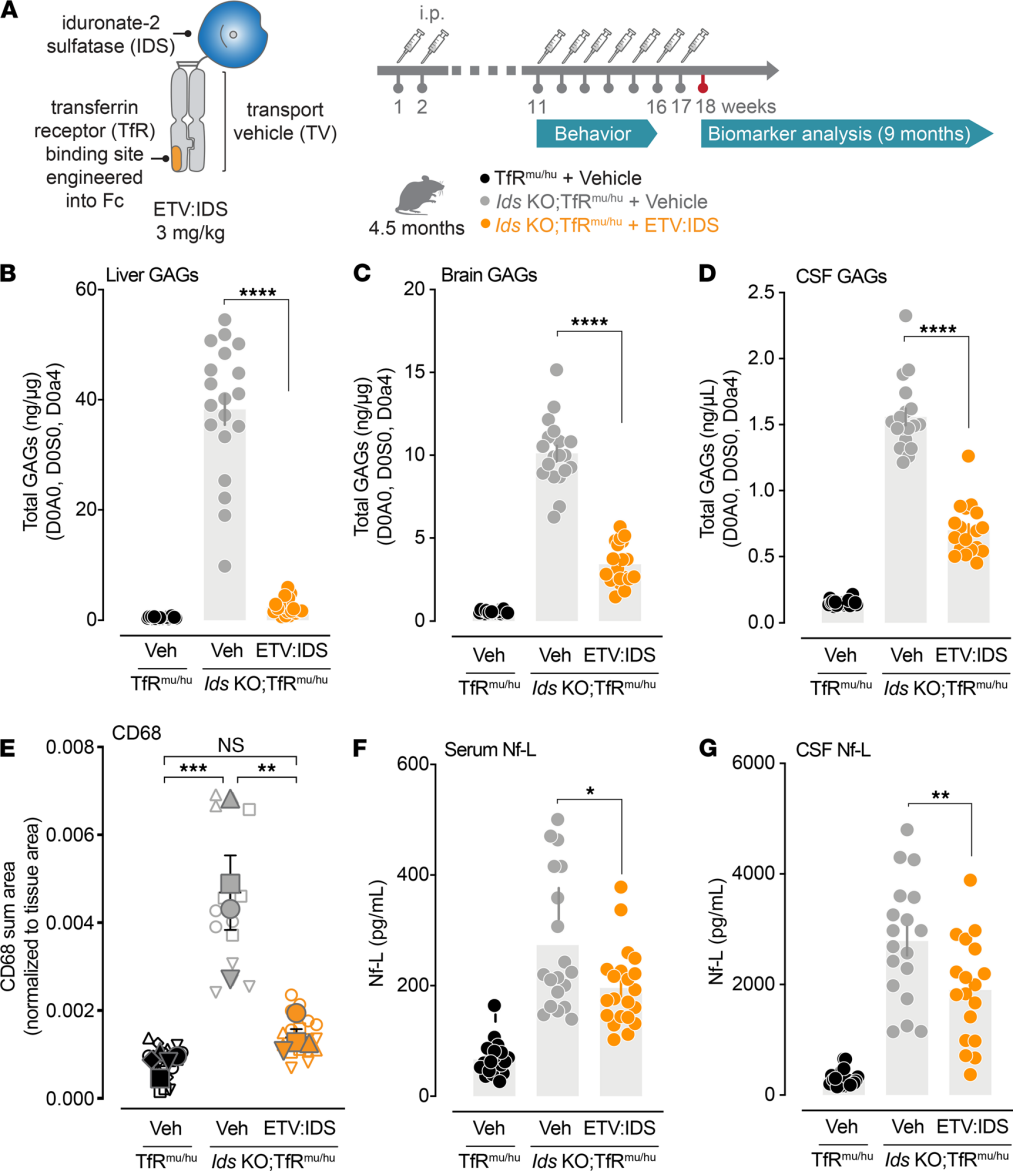
ETV:IDS reduces peripheral and CNS GAGs, microgliosis, and neurofilament light chain levels. (**A**) ETV:IDS is a fusion of IDS to the transport vehicle, a TfR-binding Fc fragment. *Ids*-KO TfR^mu/hu^ mice (*n* = 20), 4.5 months old, were administered 17 weekly doses of 3 mg/kg ETV:IDS via IP injection. Vehicle-treated TfR^mu/hu^ (*n* = 22) and *Ids*-KO TfR^mu/hu^ mice (*n* = 19) served as the nondisease and disease comparator groups, respectively. All behavioral assays were run from week 11 to week 15 of dosing, and mice were sacrificed at 9 months of age, 7 days following the last dose. GAG levels were measured in the (**B**) liver, (**C**) brain, and (**D**) CSF. (**E**) CD68 was assessed using coronal brain sections immunostained with antibodies against CD68 and imaged using a wide-field fluorescence slide scanner. Quantification of CD68 staining in the hippocampus was calculated based on the total area of detected CD68-positive cells in the region of interest (ROI) divided by the total area in the ROI. Graphs display superimposed summary statistics from 4–5 animals (solid shapes) consisting of 2–3 sections from each animal (open shapes). Each animal is coded by different shapes. The 4–5 means were then used to calculate the mean ± SEM: 1-way ANOVA with Tukey’s multiple-comparison test. Neurofilament light chain (Nf-L) levels were evaluated in the (**F**) serum and (**G**) CSF. Graphs display mean ± SEM: 1-way ANOVA with Tukey’s multiple-comparison test; *P < 0.05, **P < 0.01, ***P < 0.001, and ****P < 0.0001.

We have previously reported that ETV:IDS administered intravenously (IV) in *Ids*-KO TfR^mu/hu^ mice reduced substrate accumulation and improved lysosomal function in the CNS (11). In this behavioral efficacy study, ETV:IDS was administered by intraperitoneal (IP) injection after confirming that the pharmacokinetics (PK) and pharmacodynamics (PD) of ETV:IDS were comparable when delivered either IV or IP (Supplemental Figure 1 and Supplemental Table 1; supplemental material available online with this article; https://doi.org/10.1172/jci.insight.145445DS1). To evaluate the effects of ETV:IDS, we treated *Ids*-KO TfR^mu/hu^ mice with weekly doses of vehicle or 3 mg/kg ETV:IDS via IP injection for 17 weeks (Figure 1A). Age-matched, vehicle-treated TfR^mu/hu^ mice served as a nondisease control group. In-life serum samples were collected 4 hours after the last administration of ETV:IDS to confirm peripheral exposure with previous bridging studies at comparable dose levels following IP delivery (mean ± SD concentration 68.6 nM ± 38.2 nM).

We next evaluated GAG levels in the liver, brain, and cerebrospinal fluid (CSF), 7 days following the last dose of ETV:IDS. Total GAGs were determined as the sum of the major disaccharides derived from heparan sulfate (HS) (D0A0, D0S0) and dermatan sulfate (DS) (D0a4). As we previously reported, ETV:IDS reduced GAGs in peripheral organs, such as the liver, and lowered GAG levels in the brain and CSF (Figure 1, B–D, and Supplemental Figure 2).

Neuroinflammation is a common hallmark of neuronopathic LSDs (12) and has been reported in mouse models of MPS II (11, 13, 14) and in patients with MPS II (15–18). To determine if ETV:IDS reduces neuroinflammation, we analyzed brain tissue sections immunostained for a marker of responsive microglia, CD68. Following ETV:IDS delivery, *Ids*-KO TfR^mu/hu^ mice exhibited reduced CD68 staining in the hippocampus compared with disease controls (Supplemental Figure 3). Quantitative analysis of CD68 staining (Figure 1E) confirmed elevated CD68 signal in the hippocampus of *Ids*-KO TfR^mu/hu^ mice that was significantly reduced following ETV:IDS delivery, in line with previous published work (11).

Nf-L measured in CSF and serum is considered a sensitive marker of neuronal damage (19) that is elevated in patients with neuronopathic MPS II (15) as well as in *Ids*-KO TfR^mu/hu^ mice (11). In this mouse model, Nf-L levels in the CSF were increased as early as 3 months of age and continued to rise with disease progression (Supplemental Figure 4). We recently reported that chronic ETV:IDS treatment initiated at 2 months of age, when CSF Nf-L was elevated by approximately 2-fold in this model, prevented the age-dependent increase in Nf-L (11). Here we aimed to determine if ETV:IDS could still be effective at reducing Nf-L if treatment was initiated at 4.5 months of age, a stage in disease progression when CSF Nf-L levels are substantially more elevated, which is more representative of when treatment onset might be initiated in patients with MPS II. We found that ETV:IDS significantly reduced Nf-L levels by approximately 30% in both serum and CSF of *Ids*-KO TfR^mu/hu^ mice (Figure 1, F and G), demonstrating that ETV:IDS could exert neuroprotection despite the late treatment onset. The Nf-L changes observed in serum and CSF following ETV:IDS treatment suggest that serum Nf-L may be used as a treatment-responsive biomarker of neurodegeneration, as has been demonstrated for other neurodegenerative disorders like Batten’s disease (20) and spinal muscular atrophy (21).

### Systemic administration of ETV:IDS rescues neurobehavioral phenotypes.

In addition to severe impairment in motor skills, neuronopathic MPS II is associated with the progressive emergence of neurobehavioral symptoms, which can include impulsivity, emotional dysregulation, speech/language delays, poor attention/focus, and challenges in learning basic life skills (22, 23). The *Ids*-KO mouse model recapitulates several biological and pathological aspects of MPS II disease (11, 24). Therefore, we next investigated how these manifestations may cause functional alterations of the brain by evaluating specific aspects of mouse behavior.

**Figure 2:**
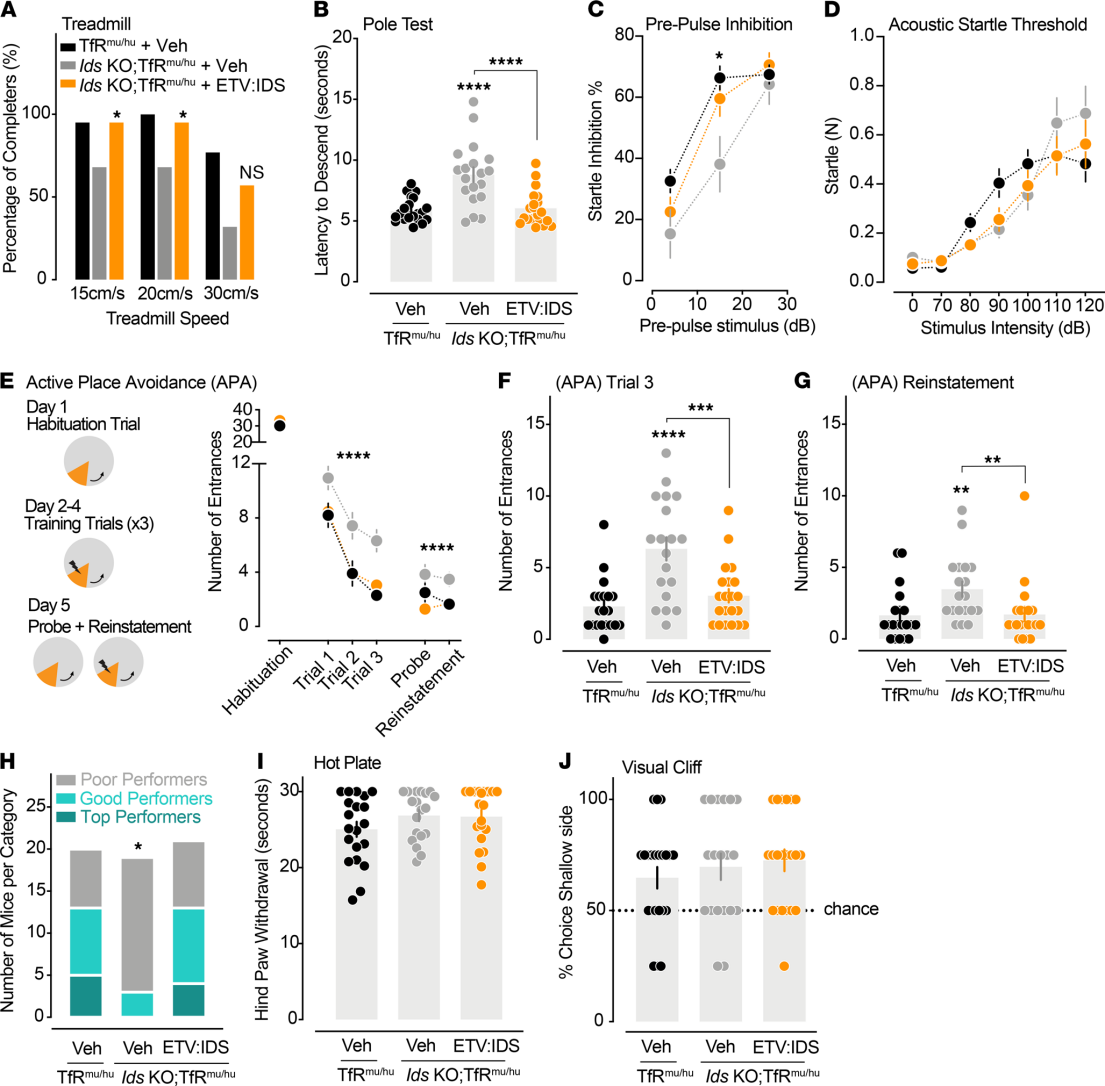
ETV:IDS normalizes neurobehavioral deficits in *Ids*-KO TfR^mu/hu^ mice. Vehicle-treated TfR^mu/hu^ (*n* = 22; black), vehicle-treated *Ids*-KO TfR^mu/hu^ mice (*n* = 20; gray), and ETV:IDS-treated *Ids*-KO TfR^mu/hu^ mice (*n* = 19; orange) underwent a battery of behavioral tests from week 11 to week 15 of dosing. (**A**) The proportion of mice that successfully completed the running trials at each treadmill speed was assessed: Fisher’s exact or χ^2^ test. (**B**) The latency to descend the pole was averaged across 3 trials: linear mixed effects model. (**C**) The level of pre-pulse inhibition of startle was assessed for each pre-pulse intensity: linear model. (**D**) The whole-body startle response (N) was assessed for each acoustic stimulus: linear mixed effects model. (**E**) In the active place avoidance assay (APA), mice were trained to avoid an unmarked aversive zone (orange) where a mild foot shock was presented upon entrance in the zone. The number of entrances in the aversive zone were measured during each phase of testing: linear mixed effects model to assess treatment effect in *Ids*-KO TfR^mu/hu^ mice. (**F** and **G**) The entrances in the aversive zone were graphed for the third and reinstatement trials: Wilcoxon’s rank sum test. (**H**) The classification of performance in the APA was based on the number of entrances in the aversive zone during the reinstatement trial; poor (>1), good (1), top (0): Fisher’s exact test. (**I**) Hind paw withdrawal was measured in the hot plate test: Wilcoxon’s rank sum test, not significant. (**J**) Visual function was assessed by evaluating an animal’s preference toward the shallow versus the deep zones in the visual cliff test: χ^2^ test, not significant. All graphs display mean ± SEM except **A** and **H**. Comparison of vehicle-treated TfR^mu/hu^ mice and *Ids*-KO TfR^mu/hu^ mice, or as indicated by brackets; *P < 0.05, **P < 0.01, ***P < 0.001, and ****P < 0.0001.

While some motor phenotypes have previously been described in the *Ids*-KO mice, only very limited data are available on potential cognitive dysfunction in this model (25–27). To evaluate multiple behavioral domains, we first compared the performance of *Ids*-KO TfR^mu/hu^ and TfR^mu/hu^ mice in several behavioral assays between 4 and 8 months of age (Supplemental Figure 5). We then tested if weekly dosing of ETV:IDS, initiated at 4.5 months of age, could reduce these behavioral deficits in *Ids*-KO TfR^mu/hu^ mice (Figure 2). *Ids*-KO TfR^mu/hu^ mice did not show any clear deficits in rotarod performance or treadmill gate (Supplemental Figure 5, A and B). However, at faster speeds on the treadmill, not all *Ids*-KO TfR^mu/hu^ mice could complete the task, indicating either a physical inability to run fast or a lack of motivation to complete a more challenging task (Supplemental Figure 5C). ETV:IDS treatment improved the performance of *Ids*-KO TfR^mu/hu^ mice, with significantly more mice completing the running task despite treadmill acceleration (Figure 2A). To further explore the motor skills and agility of these mice, we measured their performance in the pole test and found that ETV:IDS treatment also normalized the performance of *Ids*-KO TfR^mu/hu^ mice to that of nondisease controls (Figure 2B).

We next assessed if *Ids*-KO TfR^mu/hu^ mice had deficits in sensorimotor gating using the pre-pulse inhibition paradigm (PPI). The PPI assay measures the ability to inhibit the startle response to a stimulus after a weaker stimulus. We found that *Ids*-KO TfR^mu/hu^ mice showed a significant reduction in pre-pulse startle inhibition compared with control mice, demonstrating a deficit in sensorimotor gating (Supplemental Figure 5D). ETV:IDS fully rescued the startle inhibition deficit of *Ids*-KO TfR^mu/hu^ mice in the PPI assay (Figure 2C). To determine if this effect could be caused by differences in hearing sensitivity in the *Ids*-KO TfR^mu/hu^ mice, we tested these mice in the acoustic startle threshold test. *Ids*-KO TfR^mu/hu^ mice did not show any deficits in the response to broadband noise in this test (Figure 2D), suggesting that the beneficial effect of ETV:IDS observed in the PPI likely reflects an improvement of sensorimotor gating, not simply auditory sensitivity. However, the threshold test is not sensitive to the ability to perceive and respond to less intense or frequency-tuned sounds; thus, a partial contribution of changes in auditory perception to PPI performance cannot be fully excluded, especially given reports of hearing deficits in *Ids*-KO mice (28).

Finally, to determine if *Ids*-KO TfR^mu/hu^ mice had cognitive deficits, we tested their spatial learning and memory in the APA (29). The APA is a hippocampus-dependent task in which mice learn over 3 trials to use distal visual cues to avoid a fixed zone associated with an aversive stimulus (mild electric shock) while the arena is continuously rotating. The probe and reinstatement phases examine the animal’s spatial and working memory for the aversive zone in the absence or presence of the aversive stimulus, respectively (29). We found that *Ids*-KO TfR^mu/hu^ mice had impaired spatial learning, as shown by the higher number of entrances in the aversive zone compared with controls during the learning and reinstatement trials (Supplemental Figure 5, E–G). Remarkably, ETV:IDS normalized the performance of these mice to levels similar to control mice in the APA (Figure 2, E–H). To verify that the protective effect of ETV:IDS in this assay was not caused by differences in pain sensitivity or visual function, we confirmed that all experimental groups had similar pain response in the hot plate assay (Figure 2I) and were able to avoid the aversive location in the visual cliff assay (Figure 2J).

### Systemic administration of ETV:IDS corrects auricular and skeletal phenotypes.

**Figure 3:**
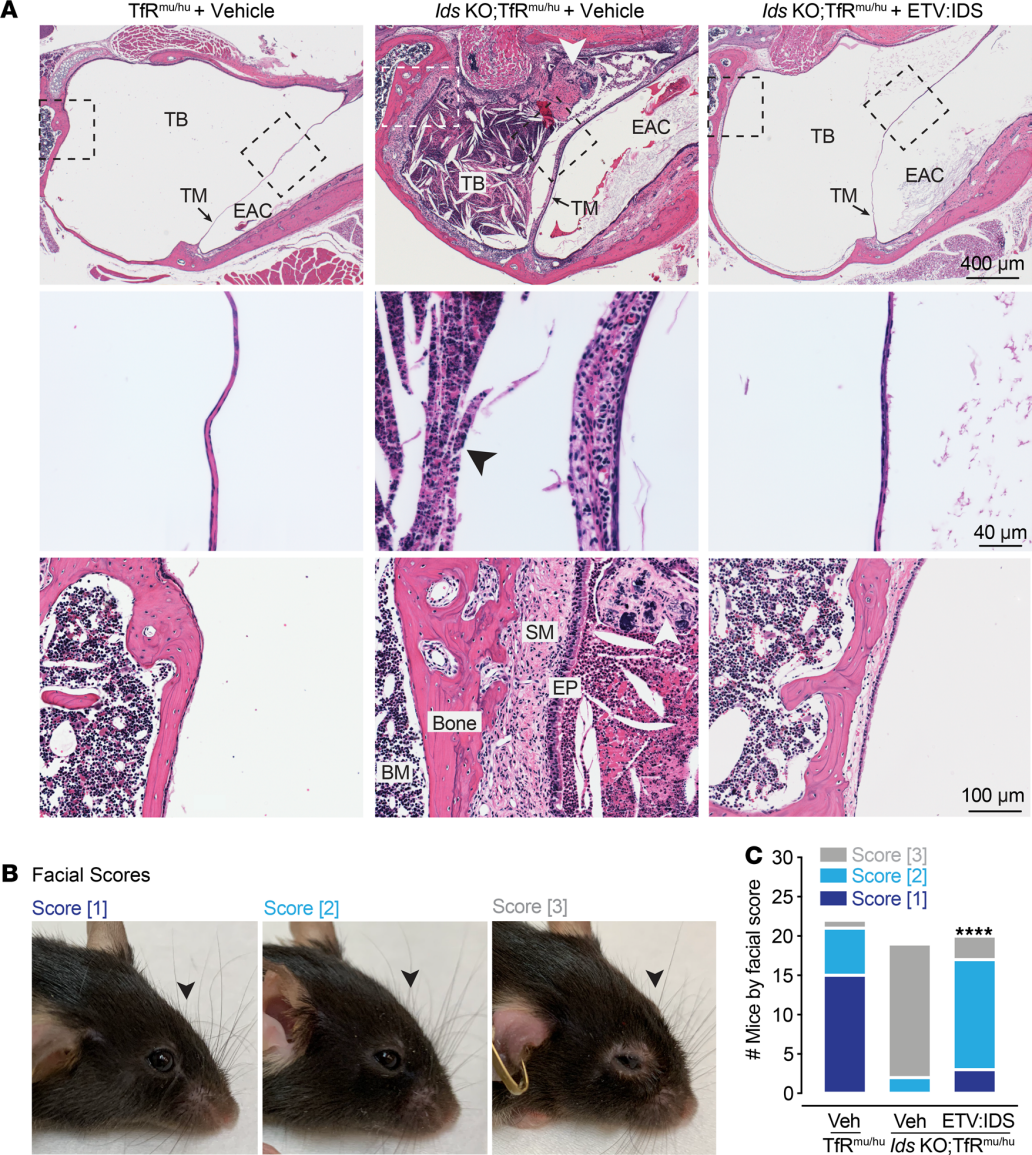
ETV:IDS improves auricular abnormalities and the facial morphology of *Ids*-KO TfR^mu/hu^ mice. (**A**) Representative hematoxylin and eosin photomicrographs of the ear canal from vehicle-treated TfR^mu/hu^ (*n* = 3), vehicle-treated *Ids*-KO TfR^mu/hu^ mice (*n* = 3), and ETV:IDS-treated *Ids*-KO TfR^mu/hu^ mice (*n* = 3). Scale bar: 400 μm. The tympanic bulla (TB) in the examined *Ids*-KO TfR^mu/hu^ mice had effusion and/or chronic otitis media as evidenced by the presence of cellular debris and exudate (arrowhead) and expansion of the tympanic membrane (TM) by inflammatory cell infiltrates (higher magnification below; scale bar: 40 μm). Higher magnification of the lining of the TB (below; scale bar: 100 μm) showed the submucosa (SM) of the TB epithelium (EP) was expanded by edema or dense connective tissue (fibrosis). Fibrous polyps were also evident (arrowheads); EAM, external auditory canal; BM, bone marrow cells. (**B**) Representative images of the severity of an animal’s shortened, broadened snout (arrowhead) and each facial score. (**C**) Graphs display the number of mice blindly scored within each category; vehicle-treated TfR^mu/hu^ (*n* = 22), vehicle-treated *Ids*-KO TfR^mu/hu^ mice (*n* = 20), and ETV:IDS-treated *Ids*-KO TfR^mu/hu^ mice (*n* = 19): Fisher’s exact test; ****P < 0.0001.

Progressive conductive and sensorineural hearing losses, resulting from GAG accumulation in the middle ear and recurrent otitis, are common in patients with MPS II (30, 31). Although there are limited clinical data on the efficacy of ERT on auditory function, data support an improvement in hearing and reduction of GAGs in the outer and middle ear of MPS II mice in response to peripherally administered ERT (28). We therefore evaluated the impact of ETV:IDS on the external auditory canal and middle ear (tympanic membrane and bulla). Compared with vehicle-treated TfR^mu/hu^ control mice, we observed marked middle ear effusion and/or chronic otitis media in the tympanic bulla of *Ids*-KO TfR^mu/hu^ mice (Figure 3A), consistent with previously described phenotypes (28). Submucosal edema and/or fibrosis with occasional polyp formation were also evident in 5 out of the 6 ear canals assessed (Figure 3A). *Ids*-KO TfR^mu/hu^ mice treated with ETV:IDS demonstrated dramatic improvement and generally displayed normal external and middle ear morphology (Figure 3A). After treatment, mild submucosal fibrosis was only observed in 1 out of the 6 ear canals assessed.

Musculoskeletal abnormalities are present in multiple MPS disorders, including MPS II (32). Some of the major manifestations on the joints and bones of patients with MPS II include disproportionally short stature, joint stiffness/contractures, thoracolumbar kyphosis, hip dysplasia, and dysostosis multiplex (32). The mouse model of MPS II displays skeletal abnormalities similar to what is observed in patients with MPS II, with skeletal changes such as sclerosis and enlargement of the bones of the skull observed as early as 4 weeks of age and progressively worsening as the animals age (24, 26). Additionally, micro-CT analysis of the femur in MPS II mice at 4.5 months of age, the age of ETV:IDS treatment initiation, showed increased skeletal bone density and bone volume (Supplemental Figure 6). Given that ERTs and hematopoietic stem cell transplantation therapies have limited beneficial impact on the skeleton if not initiated early (33), we investigated the effect of ETV:IDS on the skeletal phenotypes in older *Ids*-KO TfR^mu/hu^ mice. At 9 months of age, vehicle-treated *Ids*-KO TfR^mu/hu^ mice displayed short, broadened snouts compared with control mice, consistent with abnormal skull development (24, 26). Interestingly, the facial morphology of *Ids*-KO TfR^mu/hu^ mice treated with ETV:IDS displayed an intermediate phenotype and, in some cases, looked indistinguishable from controls (Figure 3B). At necropsy, observers blinded to treatment condition scored the facial profiles of each animal (1 to 3) based on the qualitative assessment of the severity of their shortened, broadened snouts, with a score of 3 representing severely abnormal skull development. TfR^mu/hu^ control mice had more facial scores of 1 and 2, while vehicle-treated *Ids*-KO TfR^mu/hu^ were almost all exclusively given a score of 3. *Ids*-KO TfR^mu/hu^ mice treated with ETV:IDS had more facial scores of 1 and 2 compared with scores of 3, showing an improvement in the animals’ facial abnormalities following ETV:IDS administration (Figure 3C).

**Figure 4:**
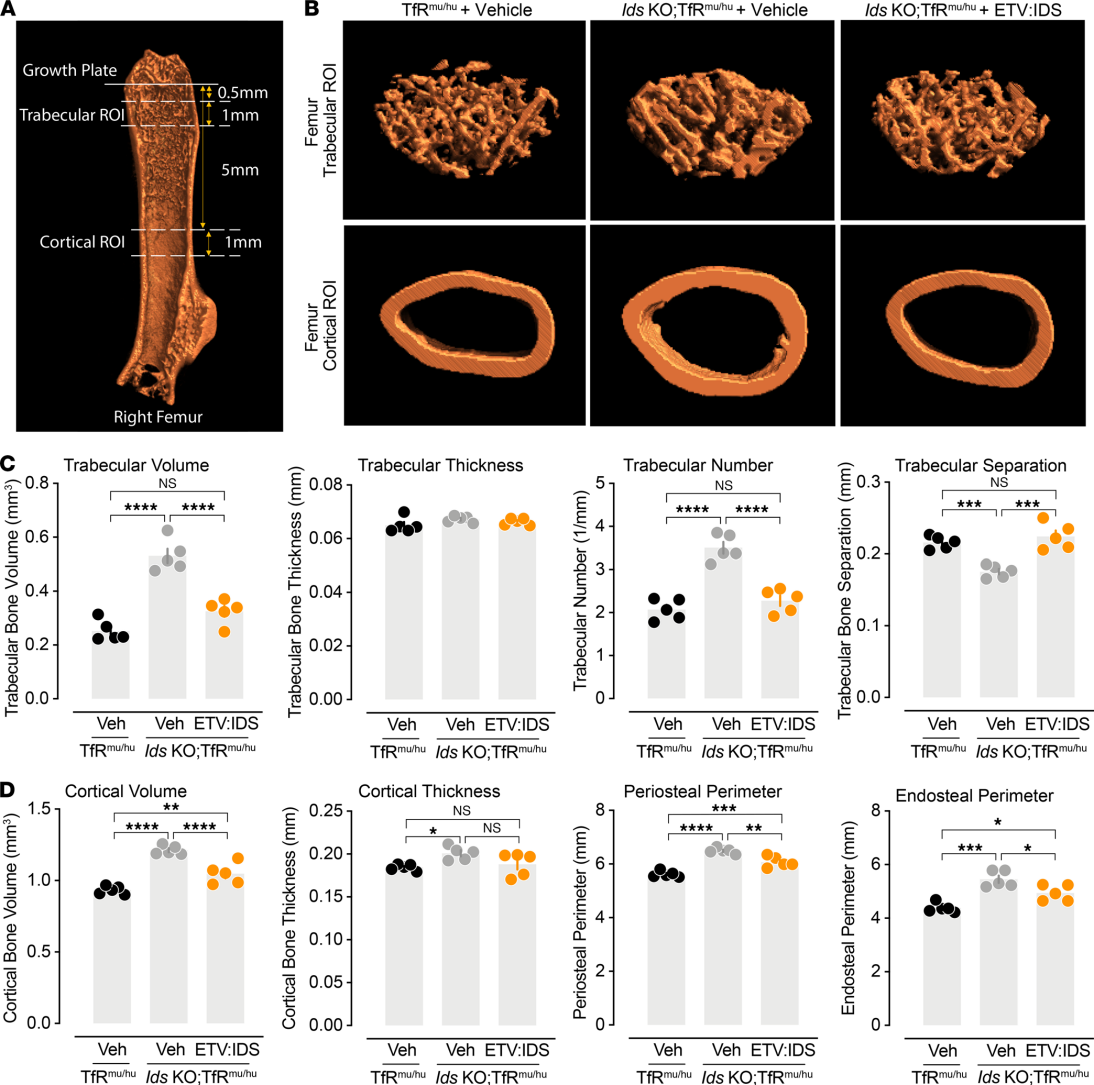
ETV:IDS improves skeletal abnormalities in *Ids*-KO TfR^mu/hu^ mice. (**A**) Representative image of the femur defining the ROIs for analysis of trabecular and cortical bone. (**B**) Representative cross-sectional images from vehicle-treated TfR^mu/hu^ (*n* = 5), vehicle-treated *Ids*-KO TfR^mu/hu^ mice (*n* = 5), and ETV:IDS-treated *Ids*-KO TfR^mu/hu^ mice (*n* = 5). Quantitative analysis from the micro-CT scans of the (**C**) femur trabecular and (**D**) femur cortical bone. Graphs display mean ± SEM: 1-way ANOVA with Tukey’s multiple-comparison test; *P < 0.05, **P < 0.01, ***P < 0.001, and ****P < 0.0001.

We further investigated the effect of ETV:IDS on skeletal phenotypes using micro-CT to acquire images of the femur and conduct quantitative image analysis of trabecular and cortical bone (Figure 4A). Compared with controls, vehicle-treated *Ids*-KO TfR^mu/hu^ mice at 9 months of age had increased trabecular density and thicker cortical bone, consistent with previously described phenotypes of long bones in this mouse model of MPS II (24). Following ETV:IDS treatment, the trabecular and cortical bones of *Ids*-KO TfR^mu/hu^ mice looked similar to those of control mice (Figure 4B). Quantitative analysis demonstrated a significant increase in the total amount of trabecular bone (volume) and a decrease in mean trabecular separation in vehicle-treated *Ids*-KO TfR^mu/hu^ mice compared with controls (Figure 4C). This is consistent with an increase in the number of trabeculae, bone mineral content, and bone mineral density, which were significantly reduced following ETV:IDS treatment and approached values observed in control mice (Figure 4C and Supplemental Figure 7A). Femur cortical bone measurements also showed that cortical bone volume, cortical thickness, bone mineral content, and endosteal and periosteal diameters were significantly increased in vehicle-treated *Ids*-KO TfR^mu/hu^ mice compared with those of controls and were reduced following ETV:IDS treatment (Figure 4D and Supplemental Figure 7B). The degree of changes observed between the *Ids*-KO TfR^mu/hu^ mice and controls, as well as in the ETV:IDS treatment group, were greater in cancellous bone (trabeculae) than in compact (cortical) bone, which is consistent with the relatively higher bone turnover rate of small trabecula. These data demonstrate that ETV:IDS can reverse skeletal disease in our MPS II mouse model given that skeletal phenotypes were already present at the time of intervention (Supplemental Figure 6).

## Discussion

Currently approved ERTs, administered peripherally, have been documented to benefit certain manifestations of disease, both in preclinical models as well as in patients with MPS II (8, 9, 34, 35). However, other aspects of disease, including neurobehavioral deficits and skeletal abnormalities, which dramatically alter the quality of patients’ lives, are not ameliorated by these therapeutic approaches. There are at least 2 potential explanations for the limited efficacy of current ERTs. First, peripherally delivered enzyme may have poor biodistribution to affected cells, making it impossible to fully restore the function of certain tissues and organs; there are a few novel ERT approaches aimed to potentially address this (36, 37). Second, treatment is often initiated late in the course of disease progression (5, 8, 38), hindering the chance of reversing damage to tissues and organs. In the current study, we demonstrate that ETV:IDS not only enhanced biodistribution of IDS in organs like the brain but also reduced neurodegeneration processes, neurobehavioral deficits, and musculoskeletal abnormalities even when treatment was initiated in adult mice.

We have previously shown that ETV:IDS enhances delivery of IDS to the brain in the *Ids*-KO TfR^mu/hu^ mouse model of MPS II through receptor-mediated transcytosis (11). The enhanced CNS delivery results in more effective reduction of GAGs compared with idursulfase in the brain and CSF as well as all CNS cell types in the brain parenchyma, including neurons, astrocytes, and microglia (11). Here, we demonstrate that enhanced brain delivery of IDS can correct functional deficits in *Ids*-KO TfR^mu/hu^ mice, improving the performance of these mice in assays testing motor and cognitive functions. Mice treated with ETV:IDS showed greater coordination and agility in the treadmill and pole tests compared with disease controls. These results suggest that ETV:IDS treatment was able to restore neural function involved in the control of complex motor behavior. However, it is also possible that the correction of the bone malformations following ETV:IDS treatment may have partially contributed to the amelioration of the motor performance in *Ids*-KO TfR^mu/hu^ mice. A recent publication showed that MPS II mice treated with a genetically engineered fusion protein consisting of a fused IDS molecule to an anti–human TfR antibody could improve performance in the Morris water maze (39). In our experiments, the *Ids-*KO mice displayed major deficits in swimming behavior (data not shown), suggesting that altered motor function is a factor in water maze performance. To get a clearer assessment of learning and memory, we selected the APA, which is less physically demanding than the Morris water maze test and can minimize the impact of motor performance on the cognitive task. ETV:IDS treatment normalized the ability of the *Ids*-KO TfR^mu/hu^ mice to memorize a spatial location in the APA, further supporting the notion that ETV:IDS can restore brain function.

In addition to the lack of current ERTs that cross the BBB and treat the neuronopathic manifestations of MPS II, the effectiveness to treat different organ/system dysfunctions, such as hearing, cartilage, and bone, is also limited, probably due to poor penetration into these less vascularized tissues (8). Joint stiffness, mobility issues, and skeletal dysplasia are major peripheral manifestations of MPS II disease. It is hypothesized that GAG accumulation impairs bone cellular function as GAG accumulation has been reported in bone and periosteal cells in some MPS animal models (40). Here we demonstrate that ETV:IDS corrected skeletal dysplasia via micro-CT imaging in our *Ids*-KO TfR^mu/hu^ mouse model of MPS II. While the mechanism of these effects on the skeletal phenotype needs to be further explored, we hypothesize that the beneficial impact of ETV:IDS on the increased skeletal bone density and bone volume in our *Ids*-KO TfR^mu/hu^ mouse model of MPS II may be a result of reducing GAG accumulation in bone and/or periosteal cells via TfR binding. We have demonstrated high TfR expression in cartilage (data not shown), supporting the proposed mechanism of normalizing endochondral ossification.

Notably, treatment in the study outlined here was initiated in mice at a stage in disease progression where neuronal damage and deficits in bone morphology were already established. This is of particular importance as the efficacy and effectiveness of ERTs in different tissues is largely dependent on the age at which treatment is started in patients with MPS (5, 8, 38). Interestingly, in the publication that showed improved performance of MPS II mice in the Morris water maze after delivery of a genetically engineered fusion protein consisting of a fused IDS molecule to an anti–human TfR antibody, Morimoto et al. (39) initiated treatment earlier (2.5 months of age) and continued it longer (8.5 months) than in our current study. Our study suggests that ETV:IDS could still improve cognitive function despite a late treatment onset (4.5 months of age) and a relatively short treatment duration (~4 months). Our findings are of particular interest because of the early deterioration of cognitive function in patients with neuronopathic MPS II (5, 6, 23). In many of these patients, cognitive function reaches a plateau around 2–4 years of life and then deteriorates as they age. Our preclinical data suggest that the damage induced by the lack of IDS on brain function is reversible and brings hope that ETV:IDS can not only halt the course of MPS II disease but also restore lost cognitive capabilities in patients. Here we also show that ETV:IDS is effective at reducing Nf-L, a sensitive marker of neuronal damage, when initiated at a stage in disease progression when CSF Nf-L levels are substantially more elevated, demonstrating that ETV:IDS could still exert neuroprotection despite the late treatment onset. Consistent with this observation, microgliosis was also normalized by ETV:IDS treatment.

While preclinical and clinical studies demonstrate that ERTs may have the potential to affect skeletal morphology with early intervention, many studies report limited improvement when initiated too late (8, 41, 42). Our data here demonstrate the ability of ETV:IDS to reverse skeletal dysplasia in our MPS II mouse model, as treatment was initiated at a stage in disease progression when deficits in bone morphology were already present. Unlike humans, longitudinal bone growth in mice continues after sexual maturity, albeit much more slowly, with peak bone mass achieved at 4–6 months of age. Bone turnover is also much faster in mice (approximately 2 weeks) compared with humans (6–9 months) (43). While this mouse model allows for the evaluation of bone effects in a short period of time following ETV:IDS treatment, in patients it could take several months of treatment before effects on the bone are observed. The totality of these data in our MPS II mouse model highlight the potential of ETV:IDS to have a therapeutic effect with later intervention and are more representative of when treatment onset might be initiated in patients with MPS II.

As new treatments for patients with neuronopathic MPS II emerge, clinical biomarkers are needed to better understand the link between CSF GAGs, markers of neurodegeneration, and motor and cognitive benefits. Here we demonstrate that normalization of neurobehavioral deficits in a mouse model of MPS II are associated with a reduction in GAG and Nf-L levels, suggesting a reduction in these biomarkers may reflect cognitive improvement with therapies that cross the BBB, such as ETV:IDS. We are also currently exploring additional fluid-based biomarkers to assess the level of neurodegeneration, gliosis, and bone disease both in animal models and in patients with MPS II, in hopes of correlating the level of these biomarkers with disease progression and treatment outcomes. The ability of ETV:IDS to restore function in multiple behavioral domains, while improving peripheral skeletal abnormalities in mice, raises the possibility that this approach could have therapeutic benefits for aspects of MPS II disease that are not fully addressed by current therapies.

## Methods

### Architecture of ETV:IDS.

All material used in this study was produced for research purposes. The expression purification and biochemical characterization of ETV:IDS were previously described (11). Briefly, ETV:IDS was expressed as a knob-in-hole recombinant fusion protein consisting of IDS fused to the N-terminus of a human Fc engineered to bind human TfR (10, 11).

### Mouse strains.

Generation of the *Ids-*KO TfR^mu/hu^ mice was previously described (11). Briefly, an *Ids*-KO mouse model on a B6N background was obtained from The Jackson Laboratory (JAX strain 024744). The TfR^mu/hu^ mouse line harbors the human TfR apical domain knocked into the mouse receptor (10). Homozygous TfR^mu/hu^ male mice were bred to female *Ids* heterozygous mice to generate *Ids-*KO TfR^mu/hu^ mice (11). All mice used in this study were males except for the PK study, which used mixed sex animals. Animals were distributed equally to each experimental group to account for differences in dates of birth.

### PK of ETV:IDS.

For the bridging PK study, ETV:IDS was administered IP or IV via the tail vein to approximately 3-month-old TfR^mu/hu^ mice (*n* = 4 per group) at doses of 0, 1, and 5 mg/kg body weight. In-life blood samples were collected at 0.25, 1, 4, and 24 hours via submandibular bleed, and terminal blood and tissues of animals were collected at 2, 8, 72, and 168 hours. For terminal sample collection, animals were deeply anesthetized via IP injection of 2.5% Avertin (MilliporeSigma). Blood was collected via cardiac puncture for serum collection and allowed to clot at room temperature for at least 30 minutes. Tubes were then centrifuged at 12,700 rpm for 7 minutes at 4°C. Serum was transferred to a fresh tube and flash-frozen on dry ice. Animals were transcardially perfused with ice-cold PBS using a peristaltic pump (Gilson Inc. Minipuls Evolution) and the brain was dissected. Brain tissue (50 mg) was flash-frozen on dry ice and processed for an IDS/IDS ELISA as described below.

### PD of ETV:IDS.

ETV:IDS was administered via IP injection to *Ids*-KO TfR^mu/hu^ mice (*n* = 20 per group) at 3 mg/kg for 17 weekly doses, and mice were sacrificed 7 days following the last dose. Vehicle-treated TfR^mu/hu^ (*n* = 22 per group) and *Ids*-KO TfR^mu/hu^ mice (*n* = 19 per group) served as the nondisease and disease comparator groups, respectively. The vehicle used for the control groups was a sodium phosphate–based buffer solution that our test article was provided in. Mice were approximately 4.5 months of age at the start of dosing and 9 months at necropsy. Sample sizes were determined based on prior studies and past experiences with the mouse model and endpoints used. The weight of each animal was recorded once a week, prior to treatment with vehicle or ETV:IDS, and treatment order was randomized weekly. For terminal sample collection, animals were deeply anesthetized via IP injection of 2.5% Avertin. For CSF collection, a sagittal incision was made at the back of the animal’s skull, subcutaneous tissue and muscle were separated to expose the cisterna magna, and a pre-pulled glass capillary tube was used to puncture the cisterna magna to collect CSF. CSF was transferred to a Low Protein LoBind Eppendorf tube and centrifuged at 12,700 rpm for 10 minutes at 4°C. CSF was transferred to a fresh tube and snap-frozen on dry ice. Blood, serum, and tissues were obtained as described above.

For the bridging PD study, ETV:IDS was administered IP or IV via the tail vein to 2- to 3-month-old TfR^mu/hu^ (*n* = 5 per group) and *Ids*-KO TfR^mu/hu^ mice (*n* = 4–5 per group) at doses of 0, 1, and 5 mg/kg for 4 weekly doses, and animals were sacrificed 7 days following the last dose.

### Tissue processing for PK analysis.

Tissue (50 mg) was homogenized in 10× volume by tissue weight cold 1% NP-40 lysis buffer (1 mL 10% NP-40 Surfact-Amps detergent solution from Thermo Fisher Scientific, 9 mL 1× PBS, 1 tablet cOmplete Protease Inhibitor [EDTA free] from Roche, 1 tablet PhosSTOP protease inhibitor from Roche) with a 3 mm stainless steel bead using the QIAGEN TissueLyzer II for 2 rounds of 3 minutes at 27 Hz. Homogenates were then incubated on ice for 20 minutes and spun at 14,000 rpm for 20 minutes at 4°C. The resulting lysate was transferred to a single-use aliquot and stored at –80°C.

### IDS/IDS ELISAs and PK analysis.

ETV:IDS was measured in serum and brain lysates using an iduronate-2-sulfatase sandwich ELISA. A 384-well MaxiSorp plate (Thermo Fisher Scientific, catalog 464718) was coated overnight with an anti-IDS antibody (R&D Systems, Bio-Techne, catalog AF2449) and blocked with Casein-PBS Buffer (Thermo Fisher Scientific, catalog 37528) the following day. Samples containing ETV:IDS were added to the plate and incubated for 1 hour at room temperature. After a subsequent wash, a biotinylated anti-IDS antibody (R&D Systems, Bio-Techne, BAF2449) was added to bind the immobilized ETV:IDS. The IDS sandwich was then detected with a streptavidin–horseradish peroxidase conjugate (Jackson ImmunoResearch, catalog 016-030-084) followed by incubation with a 3,3′,5,5’tetra-methyl-benzidine substrate (Thermo Fisher Scientific, catalog 34028). The reaction was quenched with 4N hydrosulfuric acid (Life Technologies, Thermo Fisher Scientific, catalog SS04), and the plate was read at 450 nm absorbance wavelength on a plate spectrophotometer (Molecular Devices) to determine the concentrations of analyte in the samples. Calibration standard curves were generated for ETV:IDS using a 5-parameter logistic fit with an assay range of 0.00137–1 nM. Protein sequence-derived molecular weights were used to calculate molar concentrations. Serum, liver, and brain AUC exposures for ETV:IDS were calculated using noncompartmental analysis in Dotmatics, version 4.8 (Bishop’s Stortford). Semilog and linear graphs and tabular results with standard deviations were prepared with Prism 8 (GraphPad).

### Tissue and fluid processing for GAG analysis.

Detailed methods for tissue and fluid processing for GAG analysis were previously described (11, 44). Tissue (50 mg) was homogenized in 750 μL water using the QIAGEN TissueLyzer II for 3 minutes at 30 Hz. Homogenate was transferred to a 96-well deep plate and sonicated using a 96-tip sonicator (Q Sonica) for ten 1-second pulses. Sonicated homogenates were spun at 2500*g* for 30 minutes at 4°C. The resulting lysate was transferred to a clean 96-well deep plate, and a BCA (Thermo Fisher Scientific) was performed to quantify total protein. A total of 10 μg protein lysate or 3 μL of CSF was used for subsequent HS/DS digestion. Digestion was carried out in a PCR plate in a total volume of 62 μL. Internal standard mix of HS and DS (20 ng total) were added to each sample and mixed with Heparinases I, II, and III and Chondriotinase B in digestion buffer (Iduron Ltd) for 3 hours with shaking at 30°C. After the digestion, EDTA was added to each sample, and the mixture was boiled at 95°C for 10 minutes. The digested samples were spun at 3364*g* for 5 minutes, and samples were transferred to a cellulose acetate filter plate (MilliporeSigma, MSUN03010) and spun at 3,364*g* for 5 minutes. The resulting flow-through was mixed with equal parts of acetonitrile in glass vials and analyzed by mass spectrometry as described below. Some CSF samples were not analyzed due to insufficient volume or values that were below the quantitation limit of the assay, resulting in the following sample sizes: vehicle-treated TfR^mu/hu^ (*n* = 21), vehicle-treated *Ids*-KO TfR^mu/hu^ mice (*n* = 18), and ETV:IDS-treated *Ids*-KO TfR^mu/hu^ mice (*n* = 18).

### Mass spectrometry analysis of GAGs.

Quantification of GAG levels in fluids and tissues was performed by liquid chromatography (Shimadzu Nexera X2 system) coupled to electrospray mass spectrometry (Sciex 6500+ QTRAP). For each analysis, sample was injected on an ACQUITY UPLC BEH Amide 1.7 mm, 2.1 × 150 mm, column (Waters Corporation) using a flow rate of 0.6 mL/minute with a column temperature of 55°C. Mobile phases A and B consisted of water with 10 mM ammonium formate and 0.1% formic acid, and acetonitrile with 0.1% formic acid, respectively. An isocratic elution was performed with 80% B throughout the 8-minute run. Electrospray ionization was performed in the negative-ion mode applying the following settings: curtain gas at 20, collision gas set at medium, ion spray voltage at –4500, temperature at 450°C, ion source gas 1 at 50, and ion source gas 2 at 60. Data acquisition was performed using Analyst 1.6.3 or higher (Sciex) in multiple reaction monitoring mode (MRM), with dwell time (ms) for each species, collision energy at –30, declustering potential at –80, entrance potential at –10, and collision cell exit potential at –10. GAGs were detected as [M-H]^–^ using the following MRM transitions: D0A0 at *m/z* 378.1 > 87.0, D0S0 at *m/z* 416.1 > 138.0, and D0a4 at *m/z* 458.1 > 300.0; D4UA-2S-GlcNCOEt-6S (HD009, Iduron Ltd.) at *m/z* 472.0 (in source fragment ion) > 97.0 was used as internal standard. Individual disaccharide species were identified based on their retention times and MRM transitions using commercially available reference standards (Iduron Ltd.). GAGs were quantified by the peak area ratio of D0A0, D0S0, and D0a4 to the internal standard using Analyst 1.7.1 or MultiQuant 3.0.2 (Sciex). Reported GAG amounts were normalized to total protein levels as measured by a BCA assay (Thermo Fisher Scientific).

### HS and DS calibration curves.

Pure standards for D0a4 (DS/CS), D0A0 (HS), and D0S0 (HS) were dissolved in acetonitrile/water 50:50 (*v/v*) to generate a 1 mg/mL stock. An 8-point dilution curve in PBS was generated ranging from 0.12 ng to 1000 ng. Subsequently, the internal standard D4UA-2S-GlcNCOEt-6S (20 ng) was added to each serial dilution. Samples were then boiled for 10 minutes at 95°C and then spun at 3364*g* to pellet any particulate matter. Supernatant was filtered using a 30 kDa MWCO cellulose acetate filter plate (MilliporeSigma, MSUN03010) by spinning at 3364*g* for 5 minutes at room temperature. Resulting flow-through was mixed with an equal part of acetonitrile in glass vials and run by mass spectrometry as described above.

### Fluid processing for Nf-L analysis.

Nf-L analysis of mouse CSF was previously described (11). Briefly, using Quanterix Simoa Neurofilament Light (NF-Light) Sample Diluent, CSF was diluted by 100 times and serum by 4 times before being added to the Simoa 96-well microplate. The NF-Light assay was carried out according to the Simoa NF-Light Advantage Kit instructions. Sample Nf-L levels were measured using the NF-Light analysis protocol on the Quanterix SR-X instrument and interpolated against a calibration curve provided with the Quanterix assay kit. Some CSF samples were not analyzed due to insufficient volume, resulting in the following sample sizes: vehicle-treated TfR^mu/hu^ (*n* = 20), vehicle-treated *Ids*-KO TfR^mu/hu^ mice (*n* = 18), and ETV:IDS-treated *Ids*-KO TfR^mu/hu^ mice (*n* = 17).

### Behavioral assays.

For the PD study, all behavioral assays were run from week 11 to week 15 of dosing. All testing and analysis were performed by experimenters blinded to the genotype and treatment. The rotarod test (Med-Associates, Inc.) was used to assess motor coordination and balance. The training phase, the first day of testing, consisted of 3 individual trials with an intertrial interval between 15 and 20 minutes. During the trial, mice were placed on the rotarod apparatus that was set to a constant speed of 16 rotations per minute (rpm). On the second and third day of testing, mice were placed on the rotarod apparatus with the rod rotating at an accelerated speed, from 4 rpm to 40 rpm, increasing by 3.6 rpm every 30 seconds. The mice were given 2 sessions of 3 trials each, 1 in the morning and 1 in the afternoon, for a total of 6 trials per day. All trials ended when the mouse fell off the rotarod or when 5 minutes had elapsed.

The pole test assay was used to assess motor coordination and agility. Mice were gently placed facing down atop a vertical acrylic pole 50 cm in length with a rough surface. The base of the pole was secured onto a home cage padded with bedding. Three trials were performed for a maximum of 20 seconds per trial, and the latency for the mouse to climb down, as well as the number of slides and falls, were recorded. If the mouse fell from the pole after secure placement, the animal was assigned a latency of 20 seconds. If the mouse slid less than 20% of the pole length, the latency was adjusted to 1.25 times the total latency. If the mouse slid 20%–50% of the pole, the animal was assigned a latency of 15 seconds. Latency to descend the pole was averaged across the 3 trials for each mouse.

Gait analysis was assessed using Mouse Specifics DigiGait Treadmill. Mice were placed inside the DigiGait enclosure, and the treadmill started at the initial speed of 15 cm/s. The gait analysis required 5–10 seconds of continuous running for video acquisition. Multiple attempts per animal could be required to get continuous running, with a maximum of 5 minutes acquisition time at each of the test speeds (15, 20, 30 cm/s). If a mouse was unable to keep up with the treadmill, the mouse was gently guided to the front end of the walking compartment by gently pushing the animal forward with the back panel. In addition to analyzing the gait of the animals, the number of animals in each treatment group that were able to successfully complete each speed was recorded.

Pre-pulse inhibition (Kinder Scientific) was used to assess if a weak auditory signal, shortly preceding a loud sound, can attenuate an animal’s startle response. Testing was performed in a small, isolated restraining chamber placed inside a sound-attenuating cubicle, free from external movement and noise. On the day of the test, mice were placed in the sound-attenuating chamber and given 5 minutes to acclimate to the restraining chamber and 64 dB background noise before stimulus presentations begin. Mice were then exposed to a series of acoustic startle stimuli for 20 minutes in which some stimuli were preceded by a weaker acoustic stimulus, or pre-pulse (pp), at random intervals. Trials with no auditory stimulus were also included to provide a measure of baseline activity. The test comprised a total of 80 trials randomly selected from the following list: 24 trials of 40 ms at 120 dB, and 14 trials each of the 4 dB pp 40 ms at 120 dB, 15 dB pp 40 ms at 120 dB, 26 dB pp 40 ms at 120 dB, and no stimulus trials. The interstimulus interval (ISI) was variable with a mean of 15 seconds and a range of 8–22 seconds. The percentage of pp inhibition was calculated using the following formula: 100 × ([mean of 40 ms at 120 dB trials – mean of pp trial type]/mean of 40 ms at 120 dB).

The acoustic startle threshold test was used to measure an animal’s reflexive response to sudden, loud acoustic stimuli. Testing was performed in a small, isolated chamber inside a sound-attenuating cubicle, free from external movement and noise. On the day of testing, mice were placed into the restraining chamber and given 5 minutes to acclimate to the restraining chamber and 64 dB background noise before acoustic stimulus testing began. Mice were then exposed to a series of acoustic pulses at varying intensities for approximately 15 minutes at random intervals. Trials with no auditory stimulus were also included to provide a measure of baseline startle activity. The test comprised a total of 70 trials randomly selected from the following list: 10 trials each of 40 ms at 120 dB, 40 ms at 110 dB, 40 ms at 100 dB, 40 ms at 90 dB, 40 ms at 80 dB, 40 ms at 70 dB, and no stimulus trials. The ISI was variable with a mean of 15 seconds and a range of 8–22 seconds.

The APA task (29) was used to measure visual-spatial learning and memory. Testing was performed using the BioSignal Corp. Place Avoidance system and Tracker software. Distinct black and white visual cues were placed on the walls of the room surrounding the apparatus. The grid arena was rotated at 1 rpm during the trials such that the animal must actively navigate against the rotation of the arena to avoid entry into an aversive zone. On day 1 (habituation), a stationary 60° wedge fixed to the external configuration of the spatial cues of the arena was designated as the aversive zone. Entries into this aversive zone did not result in shock delivery during the 10-minute habituation trial, and movement within the arena was used to establish a baseline of activity prior to the start of avoidance training. On days 2–4, animals were given a single 10-minute trial per day with the current source (shock) activated. Anytime the mouse entered the aversive zone, a mild shock of 0.2 mA current source lasting 500 ms was delivered every 1.5 seconds until the mouse left the zone. On day 5, the 10-minute trial was split into two 5-minute phases. The first phase was the probe test in which the current source was turned off in order to examine the animal’s pure spatial memory for the aversive zone without the presence of the unconditioned stimulus. After 5 minutes had elapsed, the current source was turned on only when the animal had clearly left the aversive zone. This reinstatement phase continued for the remaining 5 minutes of the session and provided a measure of working memory if a priming shock was delivered.

The hot plate test measured supraspinal nociception and was measured on a black anodized aluminum plate. The surface of the hot plate was heated to a constant temperature of 52°C. During testing, mice were placed in a clear cylindrical enclosure placed on top of the hot plate. The latency to respond with either a hind paw lick, hind paw flick, or jump, whichever came first, was measured. Each animal was only tested once and removed after a clear response was observed. To prevent injury, the maximum latency was 30 seconds.

The visual cliff test was used to measure an animal’s visual acuity. The apparatus consisted of 2 areas, a shallow and a deep side. The shallow side of the cliff was covered with a square, black checker, laminated pattern on the floor and walls while the deep side of the cliff was a transparent area suspended 101 cm above the floor to create the illusion of a cliff. For the step-off phase, mice were placed on a platform elevated 2.5 cm above the floor of the apparatus, directly straddling the shallow and deep side of the cliff. The test consisted of 4 trials with a maximum latency of 300 seconds. The latency to step off the platform and the direction of the step-off was recorded.

### Facial scoring.

The facial profile of each animal was assessed qualitatively at necropsy, and animals were given a score based on the severity of their shortened snouts. All analysis was performed by an experimenter blinded to the phenotype and treatment.

### Histology.

The skull base, containing the tympanic bullae, from 3 mice per group was used for light microscopic evaluation. Briefly, after the brain was removed, skulls were immersion fixed in 4% paraformaldehyde and postfixed in 70% ethanol. Skulls were decalcified in Immunocal Decalcifier Solution for 2 days, rinsed in water, and then processed and embedded in paraffin. Then 5 μm thick sections were cut at 100 μm intervals through the tympanic bullae, stained with hematoxylin and eosin, and coverslipped for light microscopic evaluation (Zeiss Axio Imager M2).

For CD68 imaging, fresh frozen brain tissue was sectioned coronally at 20 micron thickness using a Leica Cryostat (Leica CM 1950). Sections were directly mounted onto Fisherbrand Superfrost Plus microscope slides and stored at –80°C until processing for immunohistochemistry. Sections were warmed to room temperature and rinsed in 1× PBS for 3 rounds of 5 minutes, then fixed in 4% paraformaldehyde for 15 minutes. Slides were then transferred to Sequenza clips and rinsed in 1× PBS/0.05% Tween. Sections were then permeabilized in 0.5% Triton X-100 for 15 minutes and incubated in Universal Blocking Solution (1% BSA/0.1% fish skin gelatin/0.5% Triton X-100/0.1% sodium azide in 1× PBS) for 2 hours at room temperature. Sections were then incubated in primary antibody (BioRad MCA1957GA: rat anti-CD68, 1:500) prepared in Antibody Dilution Buffer (1% BSA/0.1% sodium azide in 1× PBS) overnight at 4°C. Sections were rinsed in 1× PBS/0.05% Tween for 3 rounds of 5 minutes followed by incubation in secondary antibody (Invitrogen, Thermo Fisher Scientific, SA5-10027: donkey anti-rat DyLight 550, 1:500) and prepared in Antibody Dilution Buffer for 2 hours at room temperature in the dark. Sections were then rinsed in 1× PBS/0.05% Tween for 3 rounds of 5 minutes and incubated in DAPI (Invitrogen, Thermo Fisher Scientific, D1306: 1:10,000 from 5 mg/mL stock) for 10 minutes. Sections were then rinsed in 1× PBS/0.05% Tween for 2 rounds of 5 minutes, removed from the Sequenza Clips, and quickly rinsed in 1× PBS. Sections were then coverslipped with Invitrogen Prolong Glass Antifade Mountant (Thermo Fisher Scientific) and cured overnight at room temperature.

### Image acquisition and quantification of CD68 levels.

Images of whole slide-mounted, immunostained coronal mouse brain sections were acquired using a wide-field epifluorescence slide scanning microscope (Zeiss Axio Scan Z1; Carl Zeiss, Inc.) with a 20×/0.8 NA air objective and filter sets to specifically image DAPI and DyLight 550–labeled secondary antibodies (see previous paragraph). Exposure times were held constant for each channel across all fields/sections/slides imaged. Every field for each tissue section image was collected, corrected for consistent flat-field shading artifacts, and poststitched using Zeiss Zen Blue Edition software (v. 2.6). To quantify levels of CD68 present in the brain, collected images were quantified using Zeiss Zen software. Hippocampal ROIs were manually outlined in a blinded fashion based on DAPI staining morphology. A custom-programmed analysis script was written to identify CD68-positive objects within the ROI based on local dynamic thresholding and size restriction. The script summed the total area of all identified CD68-positive objects and normalized to the total tissue area analyzed for each tissue section and ROI.

### Ex vivo bone micro-CT imaging and analysis.

All imaging and analysis were performed at Covance Laboratories Inc. At necropsy, the pelvis and hind limbs were immersion fixed in 4% paraformaldehyde in 1× PBS, then transferred to 70% ethyl alcohol for micro-CT imaging. For micro-CT imaging, a Bruker SkyScan-1176 micro-CT scanner was used (x-ray source: 90 kV/25 W, x-ray detector: 4000 × 2672 pixels). A calibration phantom was included during scanning and used for calibration of measurements after scanning. ROI selection for trabecular and cortical bone analysis in the femur was based on the American Society of Bone and Mineral Research published guidelines. The following scan parameters were used: x-ray source 60 kV/25 W, in-plane resolution 18 μm. For image analysis, the following parameters were measured from both the left and right femur for each animal and averaged: trabecular bone mineral density (BMD), trabecular bone mineral content (BMC), trabecular bone volume (BV), trabecular thickness, trabecular separation, trabecular number, cortical thickness, cortical periosteal perimeter, cortical endosteal perimeter, cortical BMD, cortical BMC, and cortical BV.

### Statistics.

Data were expressed as mean ± SEM, and statistical analysis was either performed in GraphPad Prism 8 or imported into R statistical computing software. All data were collected and processed in a randomized and blinded manner. One animal from the ETV:IDS-treated group was removed from all analysis due to misgenotyping. Any data that were below the quantitation limit of the assay were removed from the analysis. For assessment of GAG, CD68 staining, and Nf-L levels, analysis was performed using 1-way ANOVA with Tukey’s multiple-comparison test. The accelerated treadmill assay was analyzed using Fisher’s exact test when 1 or more groups had fewer than 5 animals in a condition; otherwise, the χ^2^ test was used. The latency to descend a pole in the pole test was averaged across 3 trials, and a linear mixed effects model was used to account for multiple trials conducted on all subjects. The analysis of pp inhibition was performed using a linear model. The acoustic startle threshold test was analyzed using a linear mixed effects model to account for the within-subject correlation obtained from having repeated measures of the same subject on different levels of auditory stimulus. For the active place avoidance test, a linear mixed effects model was used to account for repeated measures of the same subject at different stages of the experiment. The analysis of trial 3 and the reinstatement test of the APA task was performed using a Wilcoxon’s rank sum test. Animal performance on the APA task was analyzed by Fisher’s exact test. The analysis of the hot plate test was performed using a nonparametric Wilcoxon’s rank sum test. The analysis of the visual cliff test was performed using a χ^2^ test. Facial score analysis was performed using Fisher’s exact test. Quantitative analysis from micro-CT scans was performed using 1-way ANOVA with Tukey’s multiple-comparison test. *P* ≤ 0.05 was considered statistically significant.

### Study approval.

All procedures in animals were conducted in accordance with the ARRIVE guidelines 2.0. For the efficacy study, all procedures in animals were performed in adherence to ethical regulations and protocols approved by the University of California San Francisco Institutional Animal Care and Use Committee (protocol AN176332-03, San Francisco, California, USA). Mice were housed under a 12-hour light/12-hour dark cycle and had access to water and standard rodent diet ad libitum.

For the bridging PK study, all procedures in animals were performed in adherence to ethical regulations and protocols approved by the Charles River Institutional Animal Care and Use Committee (protocol US19011, South San Francisco, California, USA).

For the bridging PD study, all procedures in animals were performed in adherence to ethical regulations and protocols approved by the Denali Therapeutics Inc. Institutional Animal Care and Use Committee (protocol 2018.03.001).

## Author contributions

AA, RM, KSL, and PES conceived the study. MF and BT designed the methodology. JCD developed software. AA, RM, DC, CLC, YZ, and PES performed formal analysis. AA, RM, ERT, HNN, RR, JS, IL, JS, JH, DC, YC, JCD, IAL, HS, and BT investigated. AD provided resources. AA, RM, JS, IL, KSL, and PES wrote the original draft. AA, RM, AB, AGH, JMH, DD, KSL, and PES reviewed and edited the draft. AA, RM, and PES visualized data. AA, RM, TMG, MF, YZ, AB, KRH, AGH, SC, DD, KSL, and PES supervised the study.

## Supplementary Material

Supplemental data

## Version 1 10/08/2021

Electronic publication
