# Supplementary material for: Iduronate-2-sulfatase transport vehicle rescues behavioral and skeletal phenotypes in a mouse model of Hunter syndrome
Source: JCI Insight. 2021 Oct 8;6(19):e145445. doi: 10.1172/jci.insight.145445 (PMC8525587; doi:10.1172/jci.insight.145445)
Supplement: Supplemental data [file jciinsight-6-145445-s014.pdf]

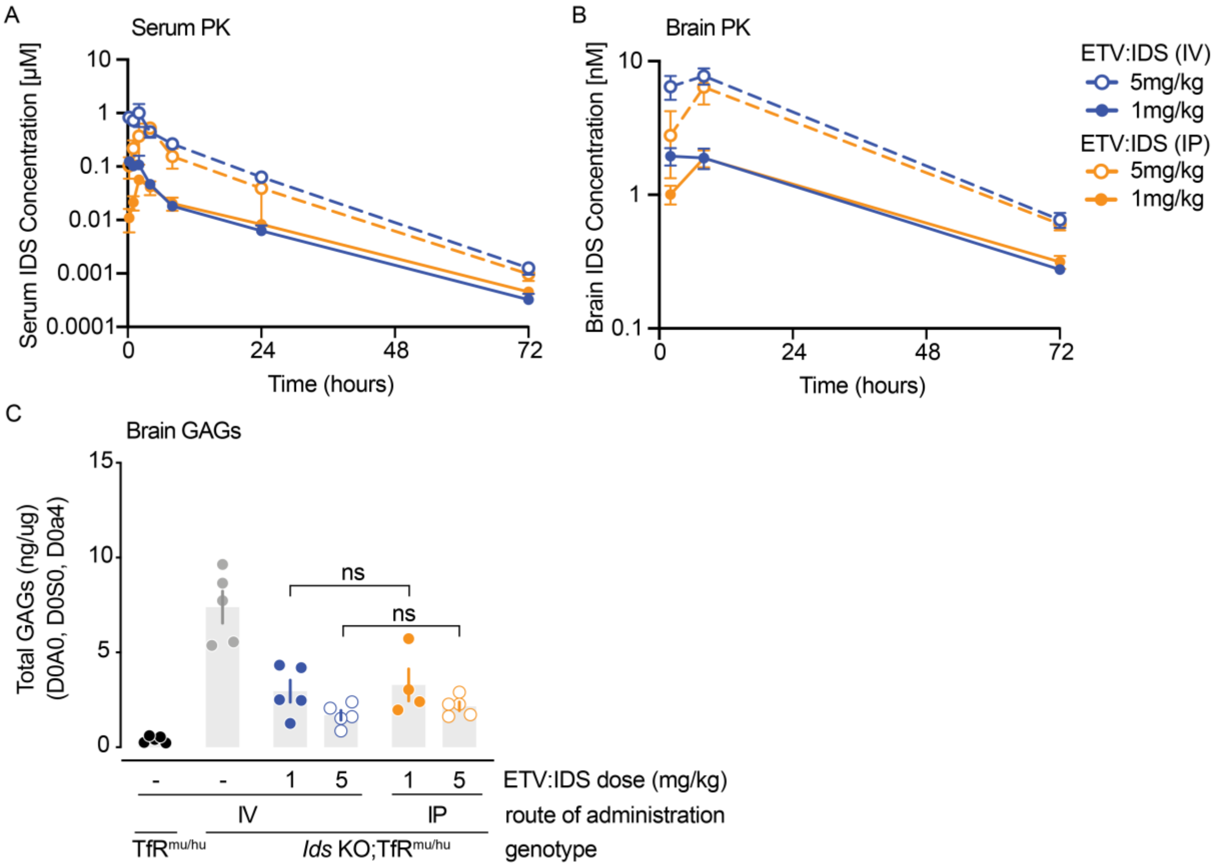

Supplemental Figure 1. Intraperitoneal (IP) delivery of ETV:IDS has a similar PK and PD response as intravenous (IV) administration. (A) Serum concentrations of ETV:IDS from TfR$^{mu/hu}$ mice were measured 0.25, 1, 2, 4, 8, 24, 72, and 168 hours after a single IV or IP dose of 1 or 5 mg/kg; *n* = 4 per group. Graphs display mean ± SD. **(B)** Brain concentrations of ETV:IDS were measured at 2, 8, 72, and 168 hours after a single IV or IP dose of 1 or 5 mg/kg; *n* = 4 per group. Graphs display mean ± SD. **(C)** GAG levels were evaluated in the brain of *Ids* KO;TfR$^{mu/hu}$ mice 7 days following treatment with ETV:IDS after 4 weekly IV or IP doses of 1 or 5 mg/kg and compared to vehicle treatment and non-diseased TfR$^{mu/hu}$ KI mice; *n* = 4-5 per group. Graphs display mean ± SEM and p values: one-way ANOVA with Tukey's multiple comparison test; ns = not significant.

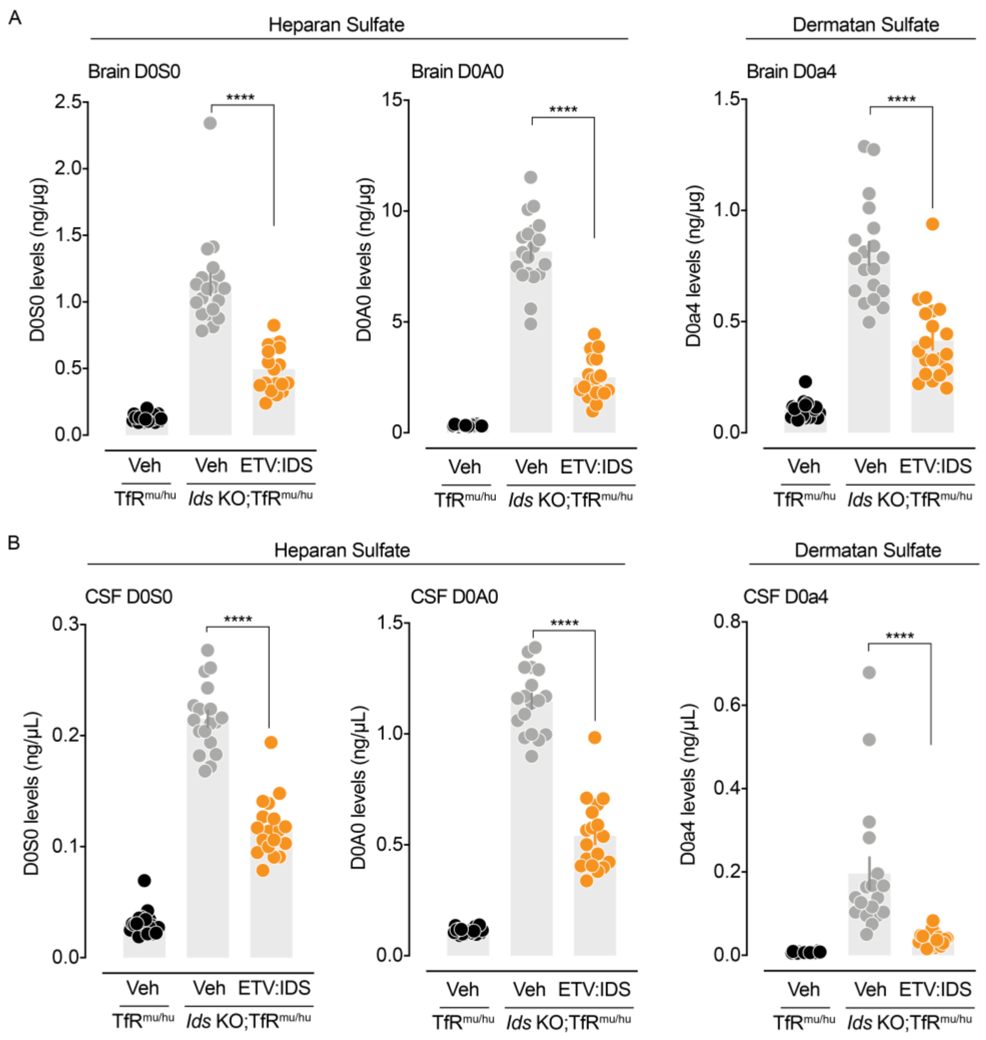

Supplemental Figure 2. Individual disaccharide analysis from the brain and CSF. Individual disaccharides derived from heparan sulfate (D0S0 and D0A0) and dermatan sulfate (D0a4) were measured in **(A)** brain and **(B)** CSF from *Ids* KO;TfR$^{mu/hu}$ mice (n = 20) administered 17 weekly doses of 3 mg/kg ETV:IDS via intraperitoneal injection. Vehicle-treated TfR$^{mu/hu}$ (n = 22) and *Ids* KO;TfR$^{mu/hu}$ mice (n = 19) served as the non-disease and disease comparator groups respectively. Graphs display mean ± SEM: one-way ANOVA with Tukey's multiple comparison test; **** $p < 0.0001$.

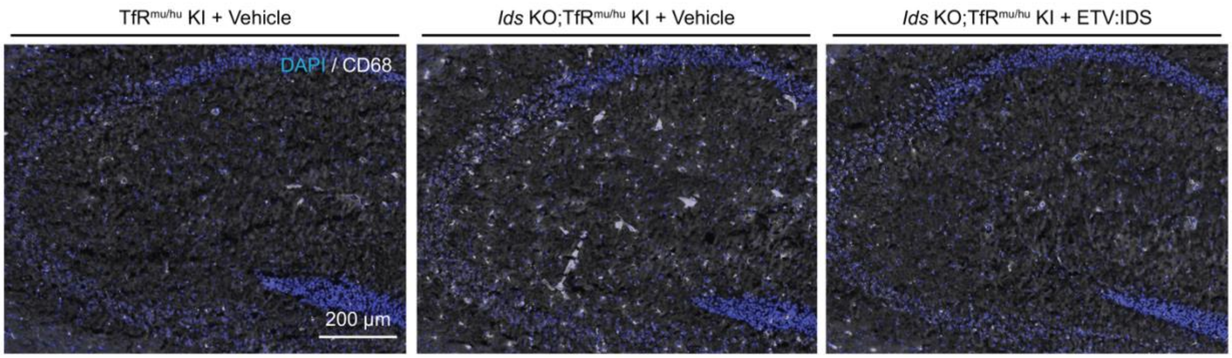

Supplemental Figure 3. ETV:IDS reduces microgliosis in the brain of *Ids* KO;TfR$^{mu/hu}$ mice. Representative brain images from vehicle-treated TfR$^{mu/hu}$ (n = 5), vehicle-treated *Ids* KO;TfR$^{mu/hu}$ mice (n = 5), and ETV:IDS-treated *Ids* KO;TfR$^{mu/hu}$ mice (n = 5). Microgliosis was assessed using coronal brain sections immunostained with antibodies against CD68 and imaged using a wide field fluorescence slide scanner. Scale bar: 200 µm.

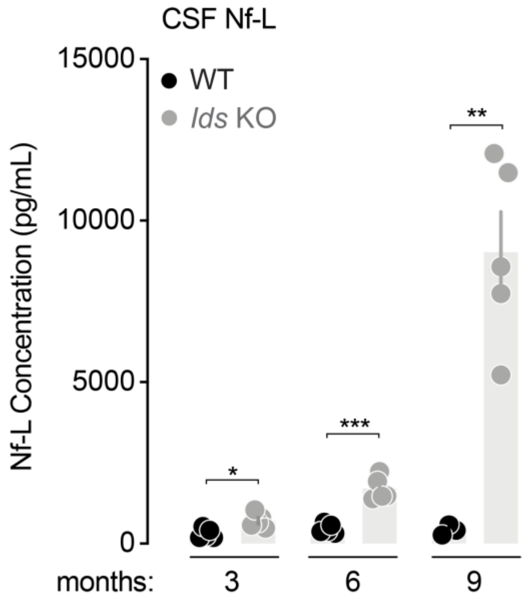

Supplemental Figure 4. Age-dependent increase in CSF Nf-L in *Ids* KO mice. (**A**) Nf-L levels were evaluated in the CSF from *Ids* KO (n = 5) and age-matched WT controls (n = 3-5). Graphs display mean ± SEM: unpaired students t-test**;** * $p < 0.05$, ** $p < 0.01$, and *** $p < 0.001$.

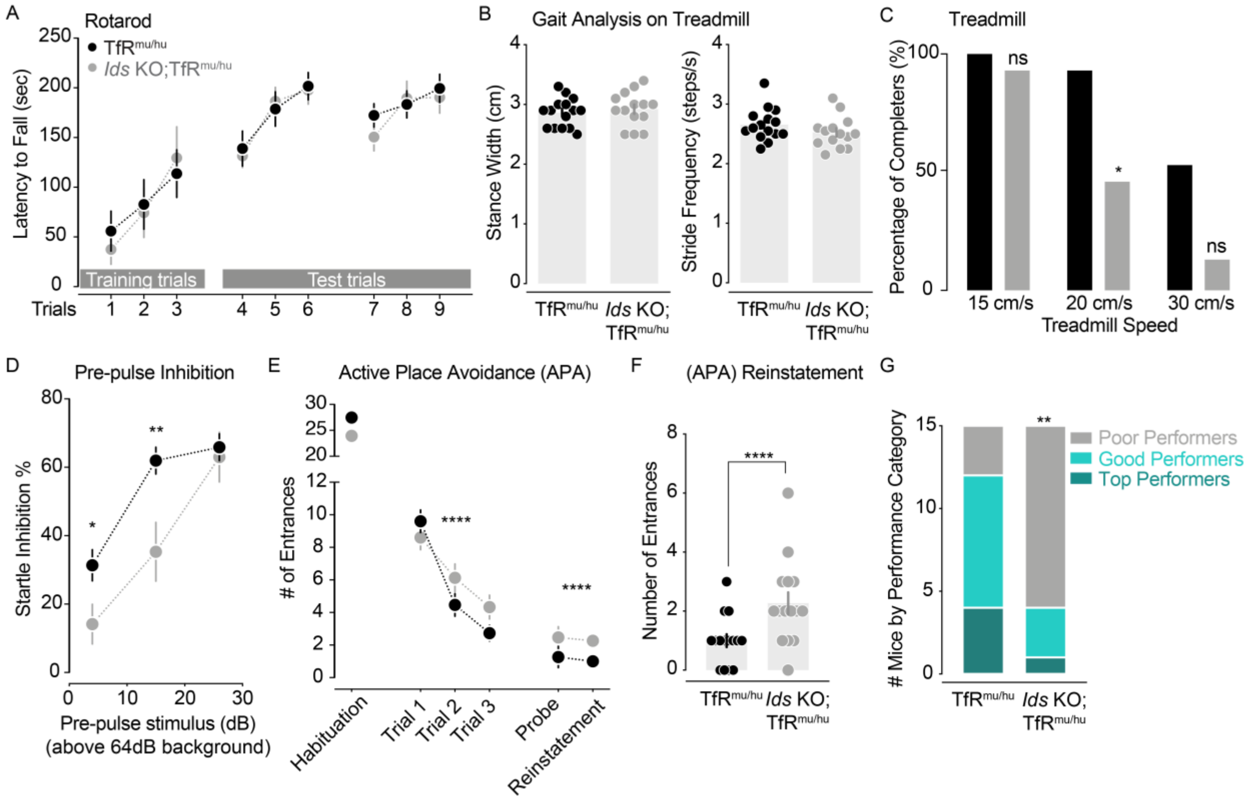

Supplemental Figure 5. Behavioral phenotypes in *Ids* KO;TfR$^{mu/hu}$ mice. We compared the performance of *Ids* KO;TfR$^{mu/hu}$ (*n* = 15; gray) and TfR$^{mu/hu}$ (*n* =15; black) mice in several behavioral assays between 4 and 8 months of age. **(A)** Using the rotarod assay, the latency to fall during the training and testing trials was assessed; linear mixed-effects model, not significant. **(B)** Gait analysis including hind paw stance width and stride frequency was assessed on the treadmill assay at 15 cm/s; linear model, not significant. **(C)** The proportion of mice that successfully completed the running trials at each treadmill speed was assessed; Fisher's exact. **(D)** The level of pre-pulse inhibition of startle was assessed for each pre-pulse intensity; linear model. **(E)** In the active place avoidance assay, mice were trained to avoid an unmarked aversive zone where a mild foot shock was presented upon entrance in that zone. The number of entrances in the aversive zone was measured during each phase of testing; linear mixed-effects model. **(F)** The number of entrances in the aversive zone during the re-instatement trial was assessed; linear mixed effects model. **(G)** The classification of performance was based on the number of entrances in the aversive zone during the re-instatement trial; poor (>1), good (1), top (0); Fisher's exact test. All graphs display mean ± SEM except contingency graphs in panels C and G. Comparison of genotype; * $p < 0.05$, ** $p < 0.01$ and **** $p < 0.0001$, ns = not significant**.**

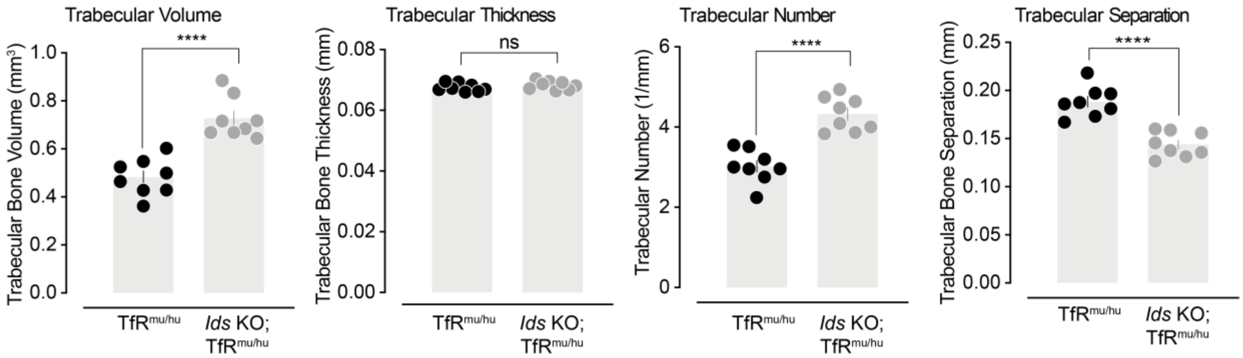

Supplemental Figure 6. Skeletal phenotypes in *Ids* KO;TfR$^{mu/hu}$ mice are present at treatment initiation. Micro-CT scans of the femur trabecular were performed from *Ids* KO;TfR$^{mu/hu}$ and age-matched TfR$^{mu/hu}$ controls at 4.5 months of age; n = 8 per group. Graphs display mean ± SEM: unpaired students t-test; **** $p < 0.0001$, ns = not significant.

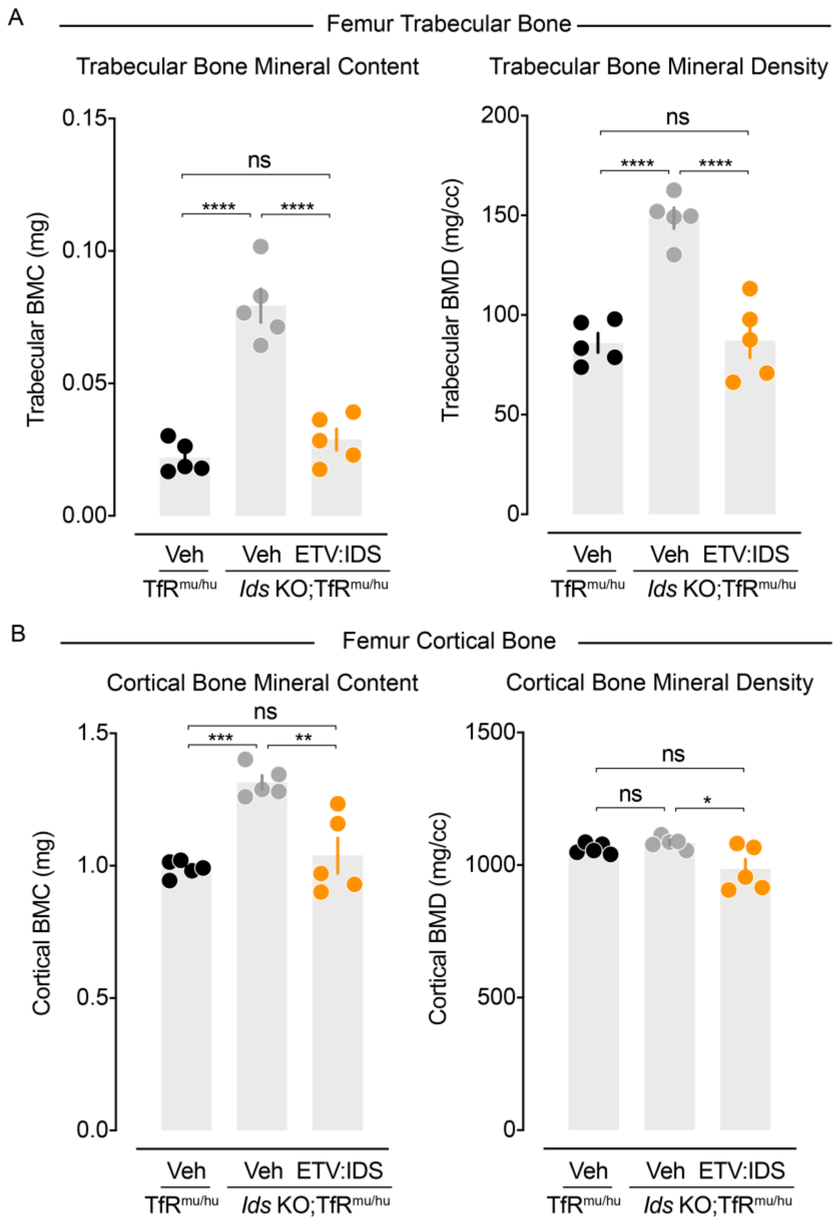

Supplemental Figure 7. ETV:IDS rescues skeletal abnormalities in *Ids* KO;TfR$^{mu/hu}$ mice. Micro-CT scans of the **(A)** femur trabecular and **(B)** femur cortical bone was performed; *n* = 5 per group. BMC = bone mineral content, BMD = bone mineral density. Graphs display mean ± SEM: one-way ANOVA with Tukey's multiple comparison test; * $p < 0.05$, ** $p < 0.01$, *** $p < 0.001$, and **** $p < 0.0001$, ns = not significant.
